# The Faith Cure Folly

**Published:** 1885-06

**Authors:** 


### The Faith Cure Folly.

The notion apparently has gone abroad that the "faith cure" folly has affected Boston literary folk generally, because a few more or less prominent writers have been quoted as devoted to it. This is not the fact, though it is true that many quite cultivated people, who would be presumed to be superior to such a shadowy and unsubstantial doctrine, have been giving it countenance during the past season, and that it is still a well-sustained Boston "craze." Possibly Miss Alcott's recently published letter, giving the benefit of her own quite thorough experiments and experiences with it, showing that "the theory which claimed to cure cancer could not help a headache," may check the "craze." But it is a question. More likely it will have its run as "crazes" in Boston generally do until the novelty is worn off. It is confined largely to women, and as this winter it has been quite fashionable in some quarters, its run naturally has been brisk. The season has been an unusually good one for such a "craze," for kindred questions, though less harmful, have been brought somewhat prominantly forward, and "psychical research" has been a popular pastime. The "faith cure" doctors have had a prosperous season while the "craze" has been on, but the regular practitioners do not appear to be at all dismayed. Indeed they look with complacency—more than this, with satisfaction—upon the growth of faith. As one of the leading of the younger doctors expressed it, the other day, the success of the physician largely depends upon the trust and faith which the patient puts in him, and there is no more difficult task than the treatment of a patient whose faith is lacking. It is quite true, as Miss Alcott says, that "every physician has cases where the mind rules the body, and works wonders with science to lend a hand;" and by and by, when the present "craze" is off, as it surely will be, sooner or later, the much discussed "faith cure" will come to be just this and nothing more.—*Boston Letter in Springfield Republican.*

ON THE SAME SUBJECT THE SCIENTIFIC AMERICAN SAYS:

It is not our purpose to deny, or even question, the verity of cures "by faith." The "mind" so acts on the body, and the brain plays so important a part in the nervous system, by which the whole organism is energized and controlled both in regard to its functions and nutrition, that it is not only quite possible, but an absolute fact, that many maladies which are not so far advanced as to be dependent upon changes in structure, or "organic diseases," may be remedied by or through the agency of the mind. We will even go so far as to affirm that a very large proportion of the ailing might be, and probably would be, sound if only they were sufficiently strongly impressed to believe themselves to be so. This influence of the mind on the body has been the stronghold of quackery from the earliest times, and "faith" is as powerful an influence for good or evil now as it has ever been. Such "miracles" as the Salvationists are working with their presage among the emotional classes, whether illiterate or well informed, have uniformly signalized the commencement of a new era in religious enthusiasm. When the first enthusiasm subsides, "miracles cease" of physico-mental necessity. The large class of so-called hysterical, cataleptic, or even epileptic affections are distinctly amenable to this influence; so are those nervous disturbances and derangements which consist wholly or chiefly in disorderly activity, as distinguished from actual disease. The mimetic maladies, of which there are always a very large number of cases, are, of course, amenable to the curative influence of faith. Outside these classes, however, stand a multitude of badly managed or misunderstood cases which only need to be placed on a new footing—it matters little what—to get well. A wondrous crowd of ignorant prejudices still hovers over many districts as to the curability of hopelessness of special diseases which are better understood and more successfully treated—on common sense principles—in the centers of knowledge.

For example, we know of localities and affections which, being associated, produce the most dire delusions as to the length of time bones usually take to unite in healthy subjects; and how coughs and other distressing maladies are, or are not, under the control of the will. In such combinations of facts and fiction, it is easy to get miracles out of such common matters as the union of the accurately applied ends of a fractured radius in three or four days! There is not a word to be said against "healing by faith." Every busy practitioner has cases under his observation that he would be heartily glad to find so powerfully affected that they could be cured even by this agency. All we are anxious to point out is that an intelligent lay press ought not to lend itself to the promulgation of nonsensical beliefs and impressions. Of course, it is true that many of the poor people who are reported to be "cured" are actually benefited, and by their faith. This is a fact, and there is no sort of reason why the benefits received should not be permanent. If the subjects of these cures are thankful to the Giver of all good, that is not a matter to make merry about. It is as it should be. We are glad of their gain, and pleased to find them moved to gratitude. Meanwhile, if these "cures" need be discussed, let the comments made be neither irreverent, offensive nor puerile. The *modus operandi* of such recoveries is perfectly well understood, and there is nothing either specially noteworthy or wonderful about them.

